# The cyclic adenosine monophosphate elevating medicine, forskolin, reduces neointimal formation and atherogenesis in mice

**DOI:** 10.1111/jcmm.15476

**Published:** 2020-08-18

**Authors:** Huifeng Hao, Xiaoyan Ma, Hong Chen, Liyuan Zhu, Zhenyu Xu, Qiaoling Li, Chuansheng Xu, Yuze Zhang, Zekun Peng, Miao Wang

**Affiliations:** ^1^ State Key Laboratory of Cardiovascular Disease National Center for Cardiovascular Diseases Fuwai Hospital Chinese Academy of Medical Sciences and Peking Union Medical College Beijing China; ^2^ Department of Integration of Chinese and Western Medicine Key Laboratory of Carcinogenesis and Translational Research (Ministry of Education, Beijing) Peking University Cancer Hospital & Institute Beijing China; ^3^ Clinical Pharmacology Center National Center for Cardiovascular Diseases Fuwai Hospital Chinese Academy of Medical Sciences and Peking Union Medical College Beijing China

**Keywords:** atherosclerosis, cAMP, endothelium, forskolin, inflammation, restenosis

## Abstract

Neointimal formation and atherogenesis are major vascular complications following percutaneous coronary intervention, and there is lack of pharmacological therapy. This study was aimed to examine the effect of forskolin (FSK), a cyclic adenosine monophosphate (cAMP)‐elevating agent, on vascular response to angioplasty wire injury and on atherogenesis in mice. Forskolin treatment reduced neointima formation at 7 and 28 days after wire injury. Early morphometrics of the injured vessels revealed that FSK treatment enhanced endothelial repair and reduced inflammatory cell infiltration. In vitro treatment of primary aortic cells with FSK, at 3‐100 μmol/L, increased endothelial cell proliferation, whereas FSK, at 30‐100 μmol/L, inhibited smooth muscle cell proliferation. FSK inhibited lipopolysaccharide‐induced leucocyte‐endothelial interaction in vitro and in vivo. In a mouse model of atherosclerosis driven by dyslipidaemia and hypertension, FSK administration increased endothelial repair and reduced atherosclerotic plaque formation, without affecting blood pressure, plasma lipids or aortic aneurysms formation. In summary, FSK, at doses relevant to human therapeutic use, protects against neointimal hyperplasia and atherogenesis, and this is attributable to its activities on pro‐endothelial repair and anti‐inflammation. This study raises a potential of clinical use of FSK as an adjunct therapy to prevent restenosis and atherosclerosis after percutaneous coronary intervention.

## 1 INTRODUCTION

Atherosclerotic cardiovascular disease is highly prevalent worldwide and life‐threatening.^1^ Percutaneous coronary intervention (PCI) using drug‐eluting stents (DES) has become the mainstay treatment for occlusive coronary artery disease. However, use of DES faces clinical challenges: neointimal hyperplasia and late stent thrombosis. Impaired re‐endothelialization represents a major mechanism underlying these complications. Agents that promote re‐endothelialization while sparing or, ideally, restraining smooth muscle cell (SMC) proliferation represents an unmet medical need for PCI treatment. Endothelial cell (EC) responses to chemical mediators, hemodynamic forces and mechanical stretch critically regulate vascular remodelling.^2^ Endothelial cell activation and/or loss of integrity promotes SMC proliferation and vascular stenosis. Improving endothelial repair bears promise for ameliorating vascular remodelling and atherosclerosis.^3^, ^4^, ^5^, ^6^

Cyclic adenosine monophosphate (cAMP) is a classical intracellular second messenger derived from ATP. Exchange protein activated by cAMP (EPAC) proteins sense cAMP and are implicated in vascular disease.^7^ In our previous study, activation of the I prostanoid (IP) receptor, a G protein‐coupled receptor (GPCR) that elevates intracellular cAMP (Gs‐coupled), inhibits SMC migration and proliferation,^8^ consistent with the function of IP in restraining neointimal formation.^9^ Other studies report that exogenous cAMP analogue inhibits SMC proliferation and balloon‐induced vascular restenosis.^10^ Recently, we report that activation of endothelial prostaglandin E_2_ receptor 4 (EP4), another Gs‐coupled GPCR, promotes endothelial repair, constrains endothelium‐leucocyte interactions and reduces intima hyperplasia.^11^ This observation suggests a role of cAMP in endothelial repair and vascular remodelling. Nevertheless, EP4 also transactivates epithelial growth factor receptor,^12^ raising a possibility that cAMP‐independent signalling might contribute to the vascular remodelling. The impact of elevating endogenous cAMP in vascular remodelling remains unknown.

Forskolin (FSK) is a potent activator of adenylate cyclase (AC), the enzyme catalysing the production of cAMP.^13^, ^14^ It increases intracellular levels of cAMP in essentially all kinds of cells, including ECs, SMCs and leucocytes. Forskolin is known for its lipolysis function^15^, ^16^ and is available as a health product. The role of FSK in vascular remodelling remains to be defined.

In this study, we report that FSK treatment promotes endothelial repair, reduces leucocyte infiltration to the neointima and ameliorates intima hyperplasia after endothelial denudation injury. It also reduces atherosclerotic plaque burden in a murine model of atherosclerosis that combines hypercholesterolaemia and hypertension. These beneficial effects are at least attributable to the effect of FSK on endothelial protection.

## 2 MATERIALS AND METHODS

See online Appendix S1.

## 3 RESULTS

### 3.1 FSK administration reduced neointimal hyperplasia after arterial wire injury

**FIGURE 1:**
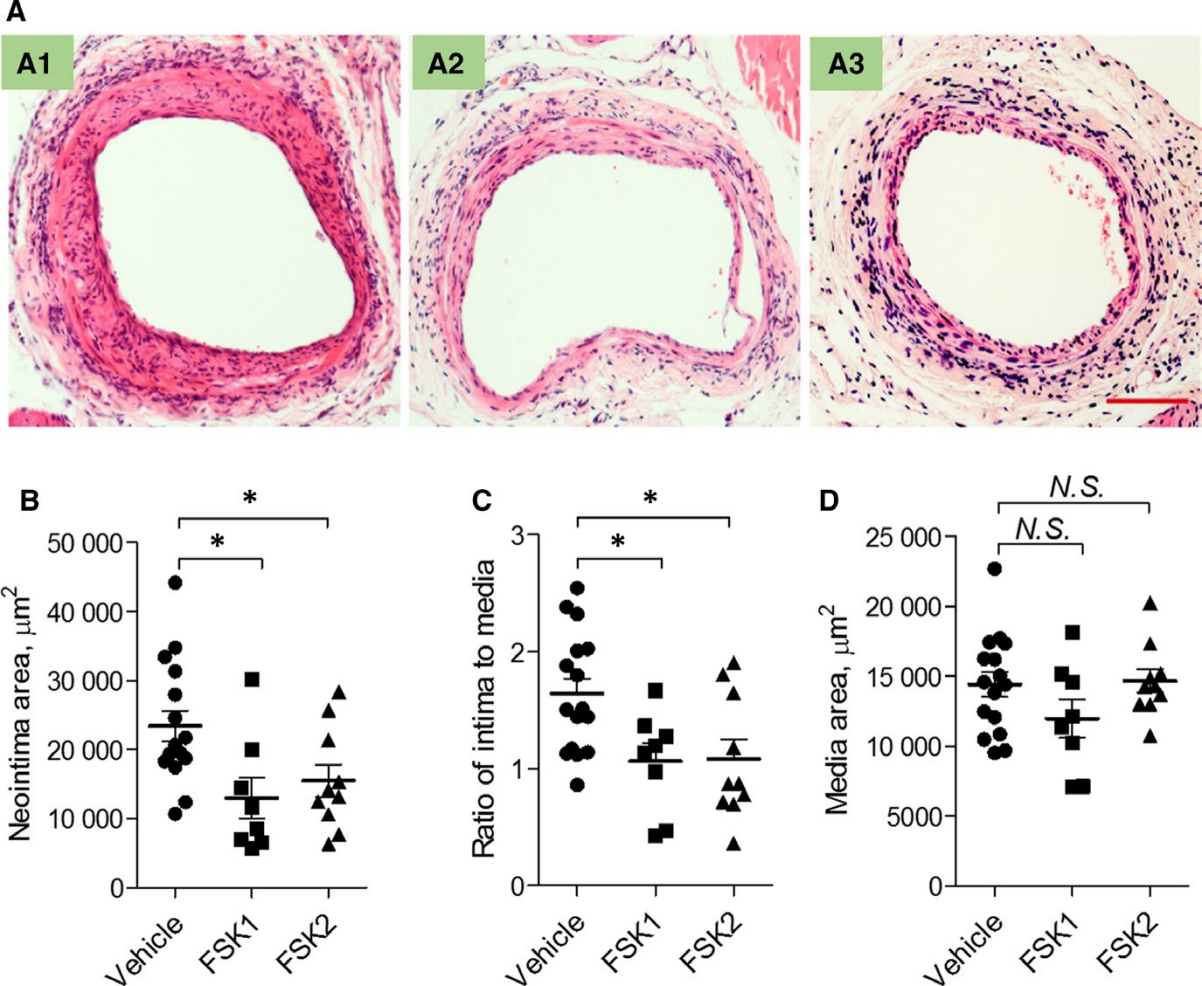
Effects of FSK on vascular responses to angioplasty wire injury. The C57BL/6 mice were subjected to wire injury of the femoral arteries, followed by being treated with vehicle or FSK for 28 d. After that, femoral arteries were harvested, sectioned and stained with H&E. A, representative H&E images of injured vessels from mice intraperitoneally treated with vehicle (A1), FSK once a day (FSK1, 2 mg/kg/Dose, A2) or FSK twice a day (FSK2, 2 mg/kg/Dose, A3) for 28 d. The scatter graphs below show the statistical results of intima area (B), ratio of intima to media area (C) and the media area (D). n = 16 (vehicle), 8 (FSK1), 10 (FSK2) mice; *P < 0.05; *t* test. Bar = 100 μm

To determine the effects of FSK on vascular remodelling, C57BL/6 mice were subjected to endothelial denudation injury at the femoral arteries by an angioplasty wire as we previously described.^5^ Vehicle, or FSK, at 2 mg/kg once or twice a day, was then administered. After 28 days, the vessels were harvested, sectioned, stained with haematoxylin and eosin (H&E), and quantified for morphometrics. FSK administration once a day (FSK1) significantly reduced neointima area and the ratio of intima to media by 44.6% and 35.2%, respectively (Figure 1A‐C). Forskolin treatment twice a day (FSK2) decreased neointima area and the ratio of intima to media by 33.7% and 34.0%, respectively (Figure 1A‐C). Forskolin treatment did not affect media area or bodyweight (Figure 1D and Figure S1).

### 3.2 FSK enhanced endothelial repair and reduced leucocyte adhesion

**FIGURE 2:**
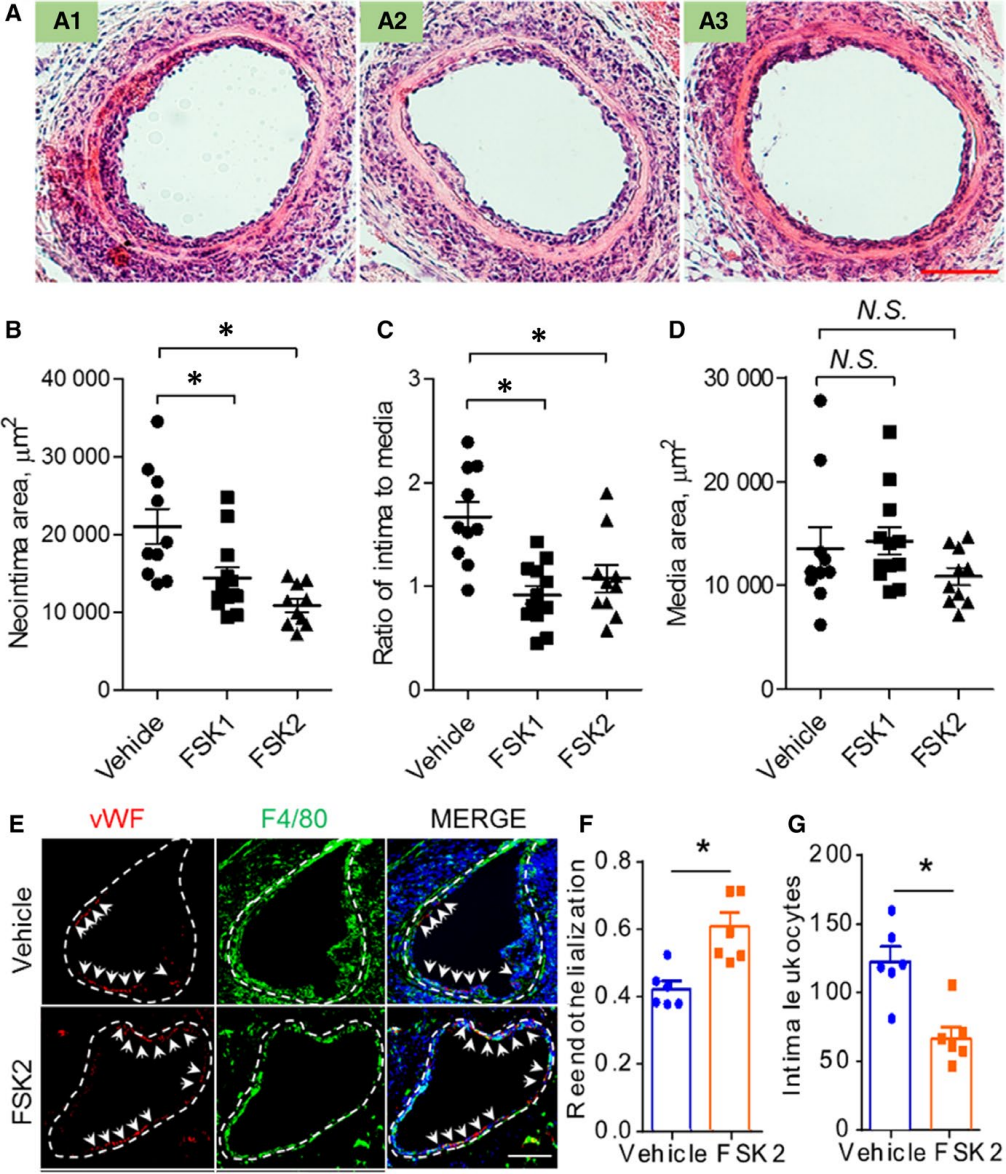
Effects of FSK on early vascular changes following wire injury. A, representative H&E images of the injured femoral arteries from mice treated for 7 d with vehicle (A1), FSK once a day (FSK1, A2) or FSK twice a day (FSK2, A3). The scatter graphs below show the statistical results of intima area (B), ratio of intima to media area (C) and the media area (D). E, representative staining of endothelial cells and macrophages in the femoral vessels isolated from vehicle and FSK2 groups. Dotted lines denote internal elastic lamina. Arrows point to vWF signals. F, quantification of endothelial coverage at day 7 of the injured vessels. G, number of intima macrophages (F4/80 positive around nuclei) in representative sections of vessels. n = 10 vehicle, 12 FSK1, 10 FSK2 for B, C and D; *P < 0.05; one‐way ANOVA with Dunnett's multiple comparison tests. n = 6 for F and G. *P < 0.05; *t* test. Bar = 100 μm

To understand the underlying mechanism, vessels from another set of experiments were harvested at day 7 after the injury to characterize early vascular pathological changes. The neointima area and the ratio of intima to media, at this time‐point, was also significantly lower in the FSK groups. Specifically, neointima area and the ratio of intima to media were reduced by 31.7% and 45.2%, respectively, in FSK1 group (Figure 2A‐C) and by 48.3% and 35.6%, respectively, in FSK2 group (Figure 2A‐C). Again, the media area was not changed (Figure 2D). Vascular endothelial cells (ECs) and leucocytes were analysed using immunofluorescent staining. FSK treatment significantly increased the endothelial coverage on the inner surface of the injured arteries compared to that of Control vessels (Figure 2E,F), indicating an enhanced endothelial repair. Meanwhile, intimal macrophages (F4/80^+^) were reduced by 45% after FSK treatment (Figure 2E,G).

**FIGURE 3:**
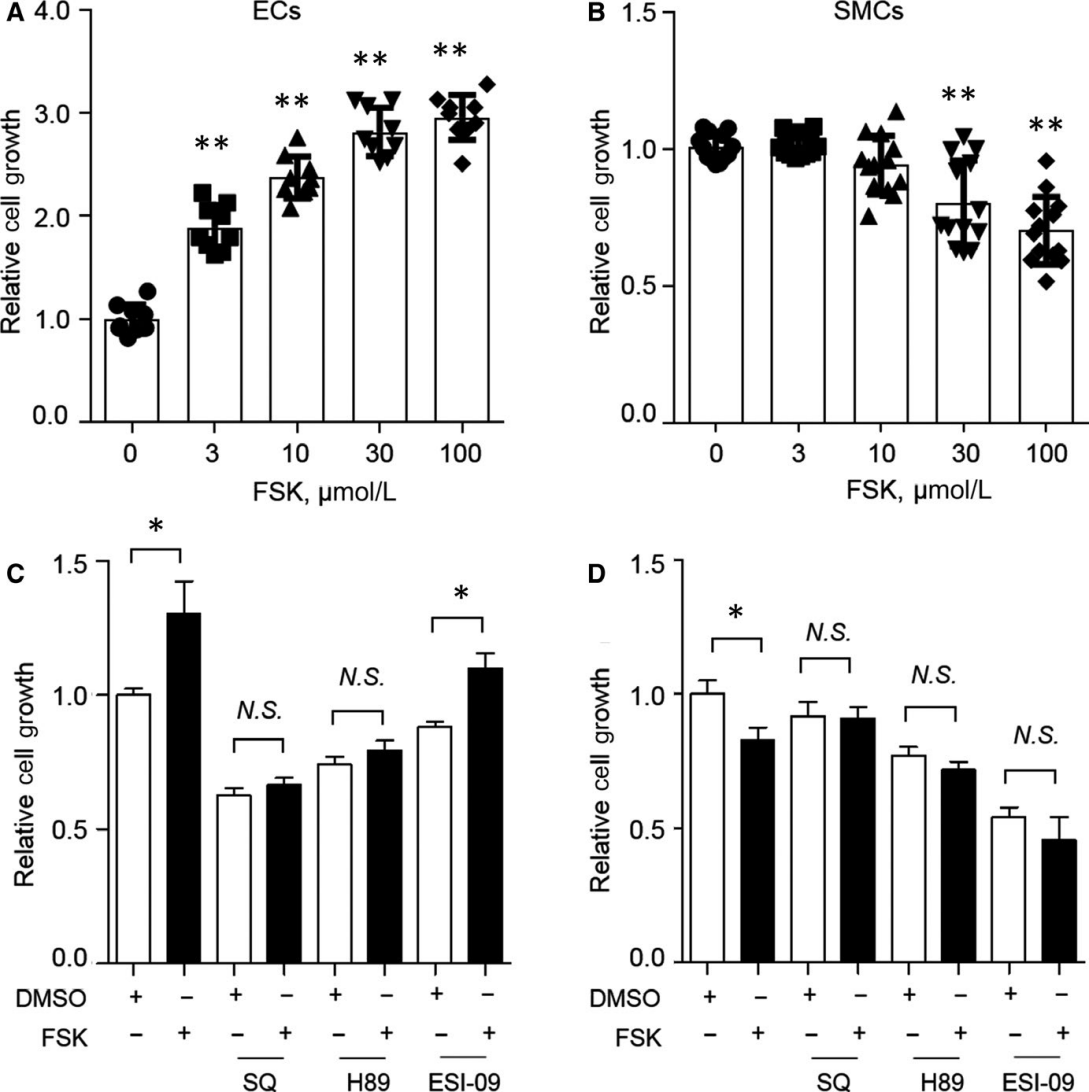
Differential effects of FSK on proliferation of ECs and SMCs. Primary ECs (A) and SMCs (B) isolated from C57BL/6 mouse aortae were treated with FSK at indicated concentrations and examined for cell proliferation as detailed in the Method section. C, In ECs, the pro‐proliferative effects of FSK (1 μmol/L) were inhibited by SQ (SQ22536—an AC inhibitor; 200 μmol/L) or H 89 (H 89 2HCl—a PKA inhibitor; 10 μmol/L) but not by ESI‐09 (an EPAC inhibitor; 10 μmol/L). D, In SMCs, the anti‐proliferative effects of FSK (100 μmol/L) became insignificant when SQ, H 89 or ESI‐09 was applied. n ≥ 9 wells from 2 or 3 independent experiments; *P < 0.05, **P < 0.01; one‐way ANOVA with Dunnett's or Bonferroni's multiple comparison post hoc tests

To further understand the underlying mechanisms, we isolated mouse aortic ECs and smooth muscle cells (SMCs) and evaluated the effects of FSK on cell proliferation. FSK dose‐dependently enhanced EC proliferation (Figure 3A). In contrast, FSK reduced SMC proliferation at high concentrations, 30 or 100 μmol/L (Figure 3B). The differential response of ECs vs SMCs is mechanistically compatible with the improved vascular remodelling following FSK treatment after angioplasty wire injury. FSK is a potent activator of adenylate cyclase (AC) leading to elevated levels of cAMP in ECs and SMCs (Figure S2). PKA and EPAC both mediate cAMP signalling. We then examined the signal pathway for the FSK regulated cell proliferation in ECs and SMCs using SQ22536 (an AC inhibitor), H 89 2HCl (a PKA inhibitor) and ESI‐09 (an EPAC inhibitor). The pro‐proliferative effect of FSK in ECs was mediated through AC or PKA, but not EPAC (Figure 3C), whereas the inhibitory role of FSK on SMC proliferation was dependent on AC, also related to PKA or EPAC (Figure 3D). The EC‐promoting effects of FSK were also compared with that of AE1‐329 (an agonist of EP4), iloprost (an agonist of IP) and misoprostol (a prostaglandin E analogue), which are all known to increase intracellular cAMP. At 10 μmol/L, FSK, AE1‐329, iloprost and misoprostol increased EC proliferation by 93%, 37%, 23% and 18%, respectively (Figure S3), indicating that FSK potently stimulated endothelial proliferation.

**FIGURE 4:**
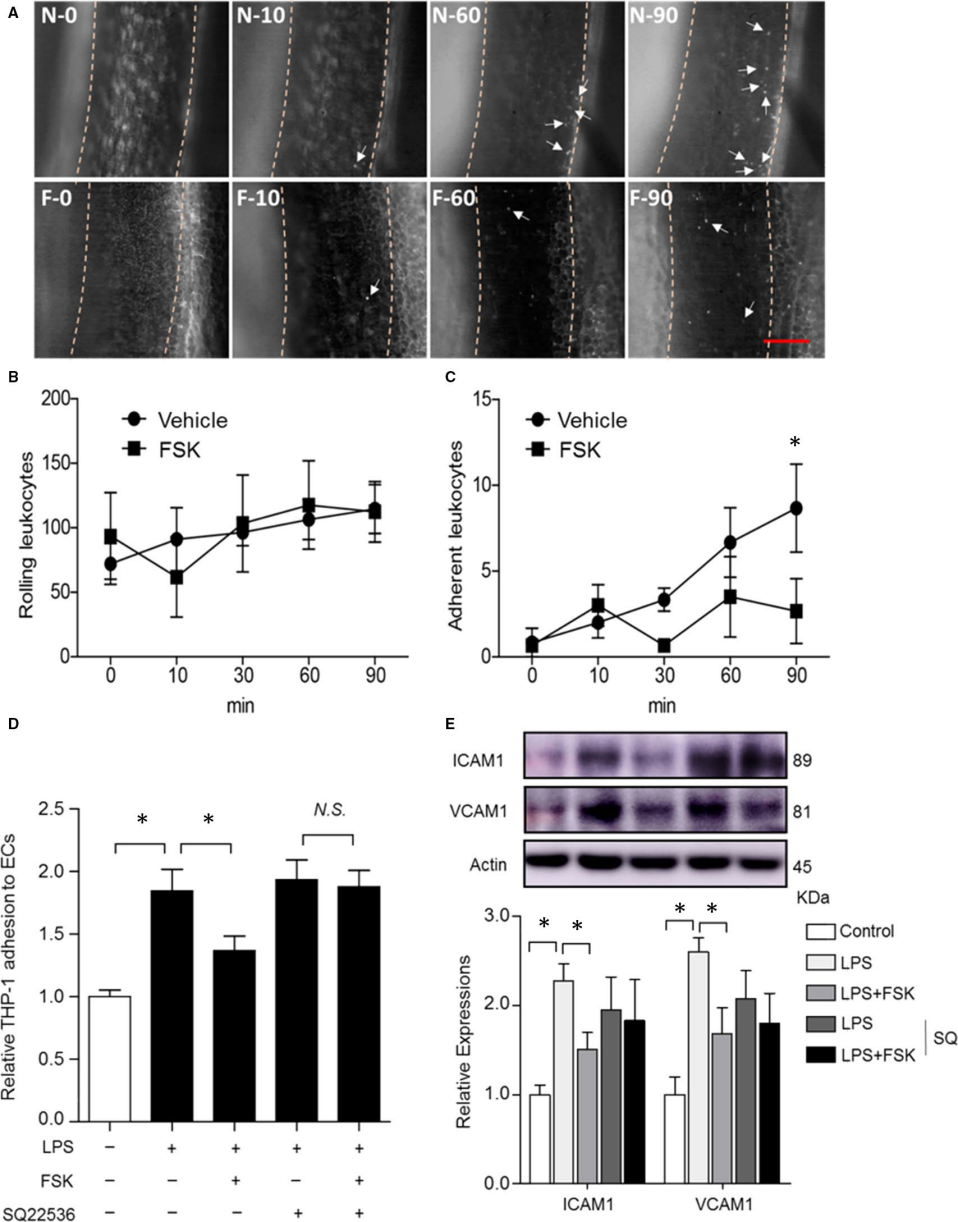
Effects of FSK on leucocyte‐endothelial interactions in vivo and in vitro. Mice were intraperitoneally administrated with vehicle (N) or FSK (F; 2 mg/kg) and, 5 min later, LPS was intravenously infused at 5 mg/kg/h to induce leucocytes rolling and adhesion. A, representative images showing rolling and adherent leucocytes at each time‐point, with the start time for LPS infusion set to 0 min. White arrows denote the adherent leucocytes. B, quantification of the rolling leucocytes at each time‐point. C, quantification of the adherent leucocytes at each time‐point. D, LPS (10 ng/mL, 2 h)‐induced THP‐1‐EC interaction was inhibited by FSK (10 μmol/L), and this effect was abolished by SQ (SQ22536—an AC inhibitor; 200 μmol/L). E, LPS (10 ng/mL, 2 h) up‐regulated endothelial expression of ICAM1 and VCAM1, which were inhibited by FSK (10 μmol/L) via AC signalling. n = 6 mice in each group in B and C, 9 in D, and 3 in E; *P < 0.05; two‐way ANOVA with Bonferroni post tests in B and C, one‐way ANOVA in D and E. Bar = 100 μm

The effect of FSK on leucocyte adhesion was directly examined both in vivo and in vitro. Mice were intraperitoneally administered FSK (2 mg/kg); 5 minutes later, lipopolysaccharide (LPS; 5 mg/kg/h) was continuously delivered intravenously for a total of 90 minutes. The rolling and adhesion of leucocytes on the endothelium were monitored using intravital microscopy at mouse femoral veins. Forskolin treatment attenuated LPS‐induced leucocyte adhesion to the endothelium (Figure 4A‐C and Video S1). The interaction of THP‐1 cells (a monocyte cell line) with ECs was also examined in vitro. Lipopolysaccharide treatment increased THP‐1 cell adhesion to ECs by 84.5%, which was significantly reduced by FSK (Figure 4D). Furthermore, Western blot result showed that LPS treatment increased endothelial expressions of VCAM1 and ICAM1, whereas FSK administration reduced the expressions of both adherent molecules (Figure 4E). Importantly, all these effects of FSK were abolished by inhibition of AC with SQ22536 (an AC inhibitor) (Figure 4D,E). Hence, FSK inhibits inflammatory cell interactions with ECs, consistent with a mechanism of anti‐inflammation in reducing wire injury‐induced neointima formation.

### 3.3 FSK administration suppressed atherogenesis in mice

**FIGURE 5:**
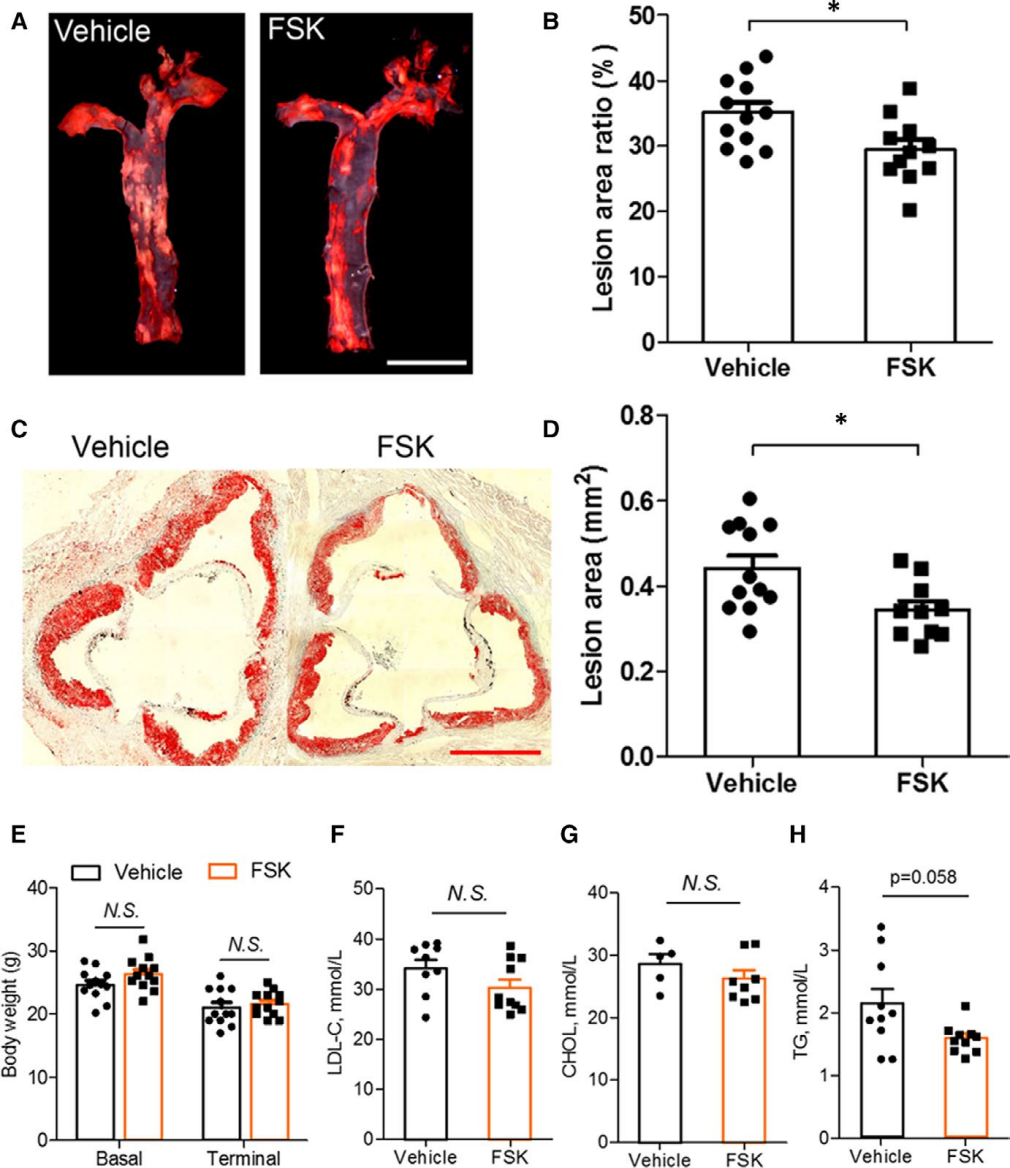
Effects of FSK on atherosclerosis in mice with both hypertension and dyslipidaemia. Eight‐week ApoE^SA/SA^ mice were initiated with HFD feeding and doxycycline administration. FSK (2 mg/kg, bid) or vehicle was applied through intraperitoneal injection. Mice were euthanized 28 d later for atherosclerosis analysis. A, representative oil Red O staining showing atherosclerotic plaque in the aorta. B, quantifications of the ratio of the areas positively stained by oil red O to the total areas of aortas. C, representative oil Red O staining of aorta roots. D, quantifications of the ratio of the areas positively stained by red oil O to the total areas of aorta roots. E, bodyweight of the mice at baseline (recorded prior to the start of this experiment) and terminal (recorded right before the mice were sacrificed). F‐H, plasma levels of low density lipoprotein‐cholesterol (LDL‐C, F), cholesterol (CHOL, G) and triglyceride (TG, H) after the mice were treated with vehicle or FSK for 28 d. n = 12 vehicle, 11 FSK in B; n = 12 vehicle, 10 FSK in D; n = 12 in E; n = 9 vehicle, 10 FSK in F; n = 5 vehicle, 8 FSK in G; n = 10 in H; *P < 0.05; *t* test

In‐stent neoatherosclerosis is an important cause for late stent failure for percutaneous coronary intervention with stenting, especially in the extended phase.^17^ We next examined the effect of FSK on development of atherosclerosis in a mouse model of severe atherogenesis (ApoE^SA/SA^). HFD feeding and doxycycline administration induce, respectively, dyslipidaemia and hypertension in ApoE^SA/SA^ mice, which leads to drastically accelerated atherogenesis (manuscript under submission). Administration of FSK for 28 days reduced atherosclerotic plaque burden in both thoracic aorta (Figure 5A,B) and aortic root (Figure 5C,D) in ApoE^SA/SA^ mice, whereas it did not influence bodyweight (Figure 5E) or plasma lipids (Figure 5F‐H and Table S1).

**FIGURE 6:**
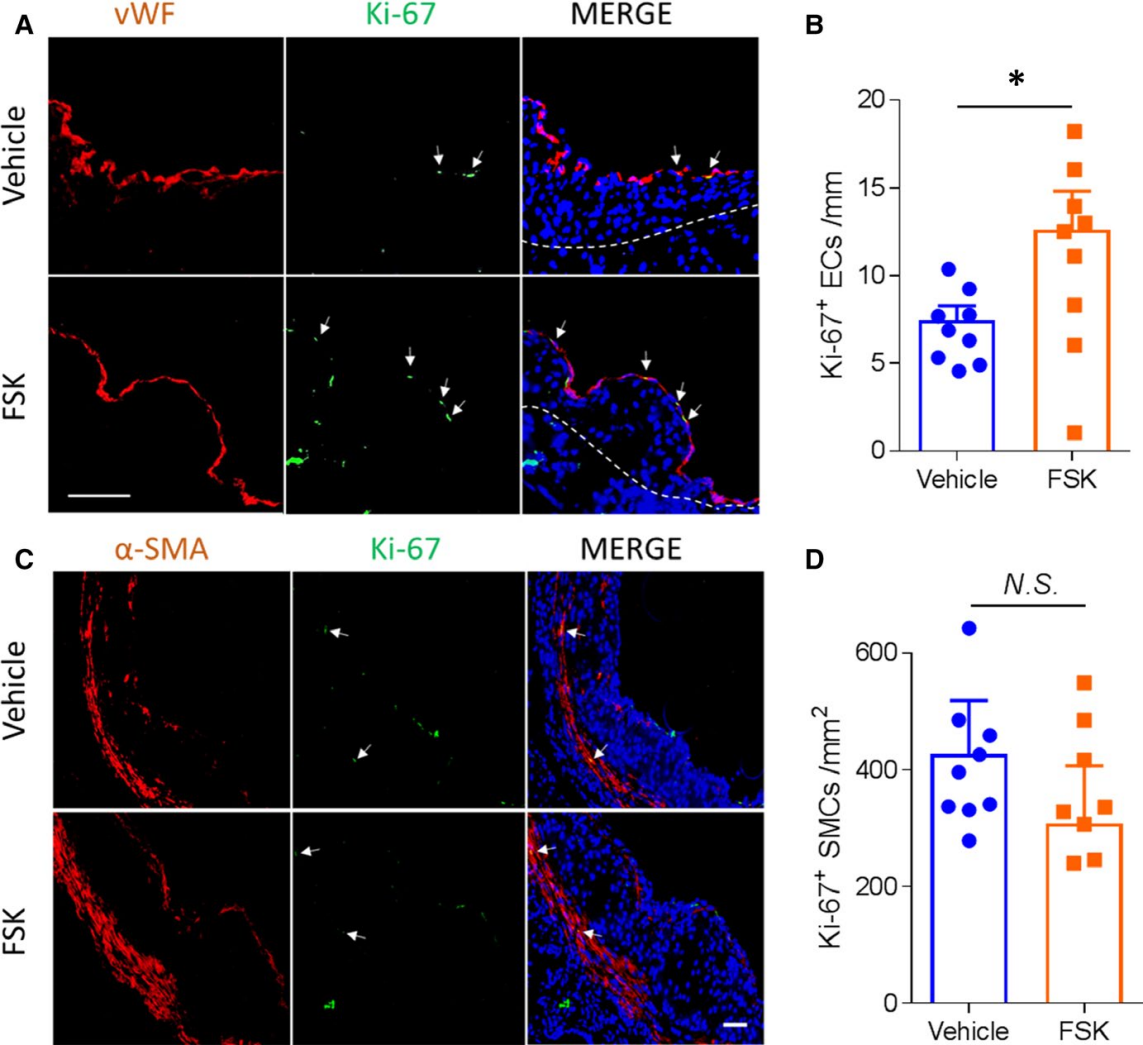
Effects of FSK treatment on EC and SMC proliferation in atherosclerotic plaques. A and C, representative Ki‐67 (a marker denoting proliferating cells) staining of ECs (A; marked by vWF) and SMCs (C; marked by α‐SMA), respectively, in atherosclerotic plaques of aortic roots. Above the dotted line is the intima layer. B and D, quantification of the proliferative ECs (B) and SMCs (D). n = 9; *P < 0.05; *t* test. Bar = 50 μm

A rapid endothelial turnover coincides with atherosclerosis‐prone vascular region,^18^ reminiscent of an active EC repairing pathophysiology. We examined the effect of FSK on proliferative activities of the lesional ECs and SMCs in aortic roots, by staining Ki‐67, a nuclear antigen denoting cell proliferation. FSK treatment strikingly increased Ki‐67‐positive ECs (Figure 6A,B), suggesting an enhanced EC proliferative/repairing activities. No significant difference was found in SMC proliferation in the plaques (Figure 6C,D).

Forskolin treatment, at the tested dose, did not modulate blood pressure, abdominal aortic aneurysms (a vascular complication of dyslipidaemia and hypertension) or survival rate (Figures S4 and S5).

## 4 DISCUSSION

Using mouse models of vascular remodelling, we report here that FSK ameliorates wire injury‐induced intima hyperplasia and attenuates atherogenesis under an exacerbated vascular injury condition combining hypercholesterolaemia and hypertension. These beneficial effects are consistent with a major impact of FSK on EC biology.

We recently reported that activation of EP4, a Gs‐coupled receptor, limits neointima formation after wire injury, which is at least in part through promoting endothelial repair.^11^ The key role of endothelial repair in neointima formation is also observed in mice with endothelial‐specific deletion of the chemokine receptor CXCR7.^5^ On the other hand, activation of the Gs‐coupled IP receptor inhibits SMC proliferation and migration, and restrains neointima formation.^8^, ^9^ These observations led us to ask whether FSK, a direct activator of adenylate cyclase to synthesize cAMP, modulates neointima formation. Indeed, FSK enhanced endothelial proliferation and repair due to elevated cAMP‐PKA signalling, and FSK restrained neointima formation (Figures 1, 2, 3). FSK suppressed SMC proliferation at higher concentrations in vitro, in a PKA‐ and EPAC‐dependent manner (Figure 3), which might also contribute to the reduced neointima formation. Furthermore, suppression of leucocyte‐endothelial interaction by FSK (Figure 4) might also underlie the protective role of FSK in the vascular response to wire injury. Hence, FSK may exert a combinatorial effect of pro‐EC repair, anti‐SMC proliferation and anti‐inflammation, which renders FSK an overall protecting effect against neointima formation. Seeking for agents that can promote endothelial healing, while suppressing SMC proliferation, has been challenging to the new drug discovery for preventing vascular restenosis post PCI. In this context, FSK might render such opportunity. How ECs and SMCs differentially respond to FSK warrants further investigation.

In‐stent neoatherosclerosis is an important cause for late stent failure for PCI with stenting.^17^ To address whether FSK modulates atherogenesis, we utilized a mouse model of atherosclerosis driven by exaggerated vascular injury from dual risk factors, dyslipidaemia and hypertension. Both these risk factors predispose to endothelial dysfunction.^19^, ^20^ Impressively, FSK treatment attenuated development of atherosclerosis under these dual challenges (Figure 5). The mechanism of FSK in ameliorating atherosclerosis might be complex, with potential modulation on the multiple risk factors and cell components of heterogeneity.^21^, ^22^, ^23^ Here, FSK treatment did not alter blood pressure, bodyweight or plasma lipids (Figure S4 and Table S1). However, FSK enhanced endothelial repairing capacity in athero‐prone region (Figure 6), consistent with its activity in pro‐endothelial proliferation (Figure 3). This protection on the endothelium is in line with the anti‐atherogenic effect of FSK, whereas SMCs did not appear to play a significant role (Figure 6). The anti‐inflammatory function of FSK (Figure 4) might also constitute to the suppressed atherogenesis. The beneficial effect on neointima formation and atherogenesis renders FSK a unique therapeutic potential for preventing vascular remodelling post PCI therapy.

FSK is a natural product isolated from the plant *Coleus forskohlii* ^24^ and is pharmacologically active in treating hypertension,^25^ thrombosis,^26^ and heart failure,^27^ which all culminate in the development or consequence of coronary artery disease. To our knowledge, the present study is the first study to evaluate the effects of FSK on vascular restenosis and atherosclerosis in vivo. The dose of FSK used in this study was 2 mg/kg, qd or bid. This is relevant to that used in the clinical conditions. The reported doses used in some clinical studies are up to 50 mg/day, which is equivalent to ～8.6 mg/kg in mice.^14^, ^16^ No relevant side effects were observed even at higher concentrations in clinical conditions.^14^ Thus, the protective effect of FSK against vascular restenosis and atherosclerosis may be extrapolatable to clinical application with a reasonable therapeutic window. It also bears a therapeutic potential to target multiple diseases under one scenario of coronary artery disease.

In summary, we demonstrate here that FSK at clinical‐relevant doses protects against vascular restenosis and atherosclerosis in mice. Mechanistically, these beneficial effects are attributable to the pro‐endothelial repair and anti‐inflammatory function of FSK (Figure S6). This study provides proof‐of‐principle evidence that FSK might be used to prevent vascular restenosis following percutaneous coronary intervention and later‐on atherosclerosis. Further clinical study is warranted to test such possibility.

## CONFLICT OF INTEREST

The authors confirm that there are no conflicts of interest.

## Supporting information

App S1

## Data Availability

The data that support the findings of this study are available from the corresponding author upon reasonable request.
