# Supplementary material for: The cyclic adenosine monophosphate elevating medicine, forskolin, reduces neointimal formation and atherogenesis in mice
Source: J Cell Mol Med. 2020 Aug 18;24(17):9638–45. doi: 10.1111/jcmm.15476 (PMC7520276; doi:10.1111/jcmm.15476)
Supplement: Supplementary file 1 — App S1 [file JCMM-24-9638-s001.doc]

**Supplemental Materials**

**1. Supplemental Methods**

**1.1 Mice**

Male C57BL/6 wild type mice, at 6-8 weeks old, were used for the wire-injury studies and the leukocyte adhesion studies. For atherosclerosis studies, 10-week old male gene-modified mice (ApoE^SA/SA^) were used, which are deficient in ApoE gene and with SR-BI knockdown and inducible angiotensin II expression (by doxycycline administration). In this study, ApoE^SA/SA^ were feed with high fat diet (HFD) containing 21% fat and 0.2% cholesterol and water containing doxycycline (1 mg/ml, protected from the light). All animal protocols complied with all relevant ethical regulations and were approved by the Institutional Animal Care and Use Committee, the Experimental Animal Center, Fuwai Hospital, National Center for Cardiovascular Diseases, China.

**1.2 Wire-injury study**

To perform the wire-injury study, C57BL/6 mice were anesthetized with intraperitoneal injection of pentobarbital sodium, 70 mg/kg body weight. Femoral arteries were injured using an angioplasty metal wire (0.35 mm diameter; Cook Inc., IN, USA) as we previously described. To explore the effects of forskolin (FSK) (S2449, Selleck company, TX, USA) on vascular remodeling, mice were randomly divided into three groups: (1) control group, in which the mice were intraperitoneally injected twice a day with vehicle (dimethyl sulphoxide (DMSO) in normal saline, 1:100 in volume), (2) FSK group 1 (FSK1), in which the mice were intraperitoneally injected with vehicle once a day and with FSK at 2 mg/Kg once a day, (3) FSK group 2 (FSK2), in which the mice were intraperitoneally injected with FSK at 2 mg/Kg twice a day. The administration of vehicle or FSK was initiated after the wire injury and was continued for 7 or 28 days. To determine the effects of FSK on neointima hyperplasia, arteries were harvested at 7 or 28 days after injury. The harvested vessels were all embedded in paraffin and sequentially sectioned for ~12 levels, with a 200 µm interval between adjacent levels. For each artery, the sections for all levels were stained with haematoxylin and eosin (H&E). The section with most severe neointima hyperplasia was selected to represent the injured vessel for further analysis.

**1.3 Cell study**

Primary mouse aorta endothelial cells (MAECs) were isolated as described previously. Briefly, aortas were harvested and cut into 1～2 mm^2^ sections, which were then attached to a petri dish with their luminal side facing down. The aortic segments were cultured in a DMEM medium containing 20% fetal bovine serum (FBS) and 100 µg/mL EC growth supplement for 5 to 7 days. Afterward, the ECs were passaged and cultured. The identity of ECs was confirmed using vWF immunofluorescent staining. MAECs in passages 2 to 6 were used in this study. Mouse aorta smooth muscle cells (MASMCs) were isolated and cultured as we described previously. THP-1(a human monocytic cell line) cells were obtained from American Type Culture Collection (ATCC, VA, USA) and cultured in RPMI-1640 medium containing 10% FBS.

Cell growth was determined with a cell counting kit-8 (CCK-8) (Yeasen, Shanghai, China) as we described previously. Briefly, MAECs in a 96-well flat-bottomed plate were firstly cultured in the medium containing 3% FBS for 6 to 8 hours, and then in a 3% FBS medium containing indicated reagents for another 48 hours. Finally, the culture medium was replaced with the medium-CCK-8 mixture for ～4 hours. Cell proliferation was then determined by examining the absorbance of the medium at a wavelength of 450 nm, using a microplate reader (Tecan, Hombrechtikon, Switzerland). To determine MASMC proliferation, cells were pre-starved in FBS-free serum for 24 hours, and then cultured in a medium containing 1% FBS.

The interaction between leukocytes and ECs was examined using THP-1 cells. ECs cultured in 96-well plates were treated with LPS (10 ng/mL) for 2 hours, with co-treatment of vehicle or FSK (10 μmol/L). Next, ECs were co-cultured with THP-1 cells that were labeled with CFSE (5 μg/mL, Selleck, TX, USA), for 30 mins. After three brief washes, the adherent THP-1 cells were determined by examining the inflorescent density under an excitation wavelength of 496 nm and an emission wavelength of 551 nm.

**1.4 Mouse atherosclerosis study**

Ten-week old male ApoE^SA/SA^ mice were randomly divided into two groups, control group (Vehicle) and FSK group (FSK2: 2 mg/Kg via intraperitoneal injection twice a day). Vehicle or FSK was administrated immediately after the mice were administered with HFD, which contains 21% fat and 0.2% cholesterol, and with Dox (1 mg/ml). Twenty-eight days later, the mice were sacrificed by intraperitoneally overdosing pentobarbital sodium (100 mg/Kg) and then perfused with 4% paraformaldehyde (PFA) via left ventricle. The hearts and aortas were then collected and fixed in 4% PFA.

Thoracic aortas and aortic roots were used for quantification of atherosclerotic plaque burden, according to previously described protocols with modifications. Briefly, thoracic aortas were cleaned off perivascular tissues, cut open, and were *en face* stained with oil red O. Percent of oil red O stained area in the total vessel area was used to represent lesion size for each individual mouse. Hearts were transversely cut off at the middle level of ventricles. The upper parts were kept in 20% sucrose solution overnight, then embedded in OCT, and frozen at -80℃. Transverse cryosections of the heart were made from the ventricle toward the aorta root. Four levels of aortic root cross sections with an interval of 40 µm between levels were collected. Sections from each level were then stained with oil red O. The maximal stained area of the aortic root sections from four levels was used to represent individual animals for comparison.

Oil red O staining for aortas and frozen sections was performed following same protocol. Briefly, the aortas and sections were stained with oil red O for 30 minutes with two brief washes using 80% methanol before and immediately after oil red O staining. Images were captured using a Zeiss light microscope (AXI0; Zeiss, Oberkochen, Germany), and analyzed using Image-Pro Plus 6.0 software (Media Cybernetics, MD). During the whole process of tissue harvest and data collection, the operator was blind to the grouping information.

**1.5 Quantification of Aneurysms**

Abdominal aorta aneurysms (AAA) were analyzed after aortae were isolated from the mice and cleaned of perivascular tissues, as previously described.

**1.6 Measurement of blood pressure**

Mouse systolic blood pressure was measured using a tail-cuff method (iiTC Life Science, Woodland Hills, CA, USA) as reported previously.

**1.7 *In vivo* analysis of leukocyte adhesion**

After mice were anesthetized with intraperitoneal injection of pentobarbital sodium at 70 mg/kg, mouse femoral veins were exposed, and visualized using an upright microscopy equipped with a camera connected to a computer. To examine the effect of FSK on leukocyte-endothelial interactions, FSK (2 mg/Kg) or vehicle was intraperitoneally injected to mice, and, 5 minutes later, continuous perfusion of lipopolysaccharide (LPS; 5 mg/Kg/h) was initiated. The rolling and adhesion of the leukocytes were monitored and recorded at the start of LPS treatment (0 min), and 10, 30, 60, 90 minutes of LPS perfusion. The time-laps recorded video was used to analyze the numbers of rolling and adherent leukocytes by colleagues in a blind manner. The leukocytes kept static for no less than 15 seconds were defined to be adherent leukocytes.

**1.8 Immunofluorescent staining**

Immunofluorescent staining for paraffin sections was performed following the same protocols that we described previously. Frozen sections were incubated with 0.25% trypsin for antigen repair, followed by blocking non-specific sites with goat serum containing 0.3% of triton. The remaining procedures for staining frozen sections and paraffin sections were same. Briefly, the sections were incubated with primary antibodies at 4°C overnight, then stained with specific secondary antibodies at room temperature for 3 hours. The primary antibodies used in this study were as follows: polyclonal anti-vWF antibody (1:800; Sigma), monoclonal anti-F4/80 antibody (1:50; Abcam), monoclonal anti-α-SMA antibody (1:500; Sigma), and monoclonal anti-Ki67 antibody (1:200, eBioscience).

**1.9 Western blot**

To examine endothelial expressions of adhesion molecules, ECs were treated with LPS (10 ng/mL) plus vehicle or FSK (10 μmol/L) for 2 hours and were harvested for Western blot analysis. Cells were lysed in a RIPA buffer, followed by a centrifugation (15800 g, 10 minutes). Cell lysates were fractionated with 10% SDS-PAGE, and transferred onto polyvinylidene fluoride membranes. The membranes were then sequentially probed with primary and secondary antibodies, with each for 1 hour at room temperature. The membranes were visualized using an enhanced chemiluminecence solution (Applygen Technologies Inc, Beijing, China). The primary antibodies used were from Abcam (Cambridge, United Kingdom) with a dilution of 1000 folds.

**1.10 Statistical Analysis**

Statistical analysis was performed using SPSS Statistics 22.0 (IBM) or GraphPad Prism 5 software (GraphPad Software, Inc, San Diego, CA). Student 2-tailed unpaired t test was used for comparisons of 2 groups. One-way ANOVA analysis was made for comparisons of multiple groups. Two-way ANOVA analysis was made for the comparisons for the *in vivo* leukocyte adhesion study. Repeated measured ANOVA was used to analysis the blood pressure data. Chi-squre test was used to compare the incidence rate of abdominal aortic aneurysm (AAA). The methods for data analysis and the correspondingpost hoc tests were all indicated in the figure legends. Results are expressed as mean±SEM. Differences were considered statistically significant at P<0.05.

**1.11 References:**

[1] Hao H, Hu S, Chen H, Bu D, Zhu L, Xu C, et al. Loss of Endothelial CXCR7 Impairs Vascular Homeostasis and Cardiac Remodeling After Myocardial Infarction: Implications for Cardiovascular Drug Discovery. Circulation 2017;135:1253-64.

[2] Zhou Z, Subramanian P, Sevilmis G, Globke B, Soehnlein O, Karshovska E, et al. Lipoprotein-derived lysophosphatidic acid promotes atherosclerosis by releasing CXCL1 from the endothelium. Cell metabolism 2011;13:592-600.

[3] Hao H, Hu S, Wan Q, Xu C, Chen H, Zhu L, et al. Protective Role of mPGES-1 (Microsomal Prostaglandin E Synthase-1)-Derived PGE2 (Prostaglandin E2) and the Endothelial EP4 (Prostaglandin E Receptor) in Vascular Responses to Injury. Arteriosclerosis, thrombosis, and vascular biology 2018;38:1115-24.

[4] Wang M, Zukas AM, Hui Y, Ricciotti E, Pure E, FitzGerald GA. Deletion of microsomal prostaglandin E synthase-1 augments prostacyclin and retards atherogenesis. Proceedings of the National Academy of Sciences of the United States of America 2006;103:14507-12.

[5] Wang M, Lee E, Song W, Ricciotti E, Rader DJ, Lawson JA, et al. Microsomal prostaglandin E synthase-1 deletion suppresses oxidative stress and angiotensin II-induced abdominal aortic aneurysm formation. Circulation 2008;117:1302-9.

[6] Daugherty A, Rateri D, Hong L, Balakrishnan A. Measuring blood pressure in mice using volume pressure recording, a tail-cuff method. Journal of visualized experiments : JoVE 2009.

**2. Supplemental Figures and Figure legends**


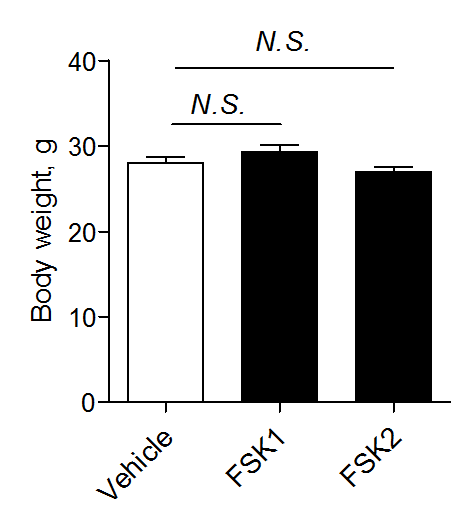


**Supplemental Figure 1. Body weight in C57BL/6 mice treated with vehicle or forskolin (FSK) for 28 days.** FSK1: Administration of FSK once a day at 2 mg/Kg/Dose; FSK2: Administration of FSK twice a day at 2 mg/Kg/Dose. n=6, One-way ANOVA.


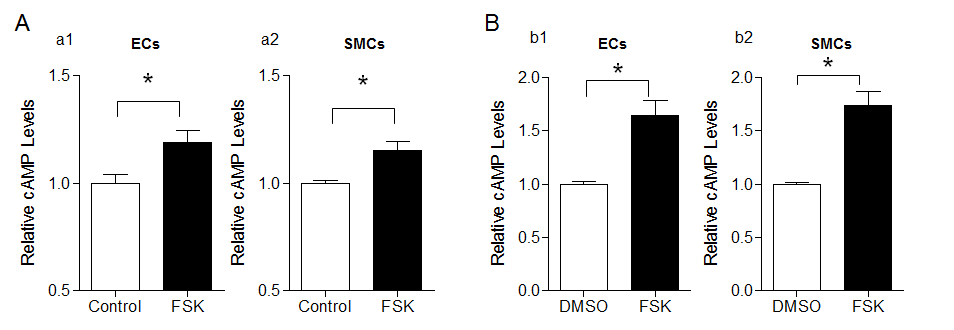


**Supplemental Figure 2.** **Effects of FSK treatment on cAMP accumulation in ECs and SMCs.** **A:** Effects of 1 hour of incubation with plasma harvested from the mice intraperitoneally injected with vehicle or FSK (2 mg/Kg) on cAMP accumulation in the ECs (a1) and SMCs (a2). The plasma was harvested 2 hours after the injection. **B:** Effects of 1 hour of incubation with the culture medium containing DMSO or FSK (30 μM for ECs, 100 μM for SMCs) on cAMP accumulation in the ECs (b1) and SMCs (b2). In A, plasma was harvested from 7 mice in each group. n=6 wells from three parallel experiments, *p<0.05, t-test.


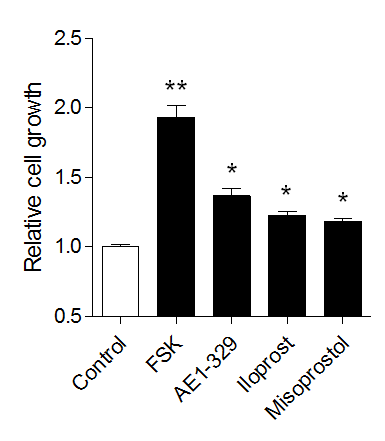


**Supplemental Figure 3.** **Effects of equal molar (10 μM) of FSK, AE1-329, Iloprost and misoprostol on endothelial proliferation.** n = 9; *p<0.05, **p<0.01; One-way ANOVA with Dunnett's Multiple Comparison post hoc tests.


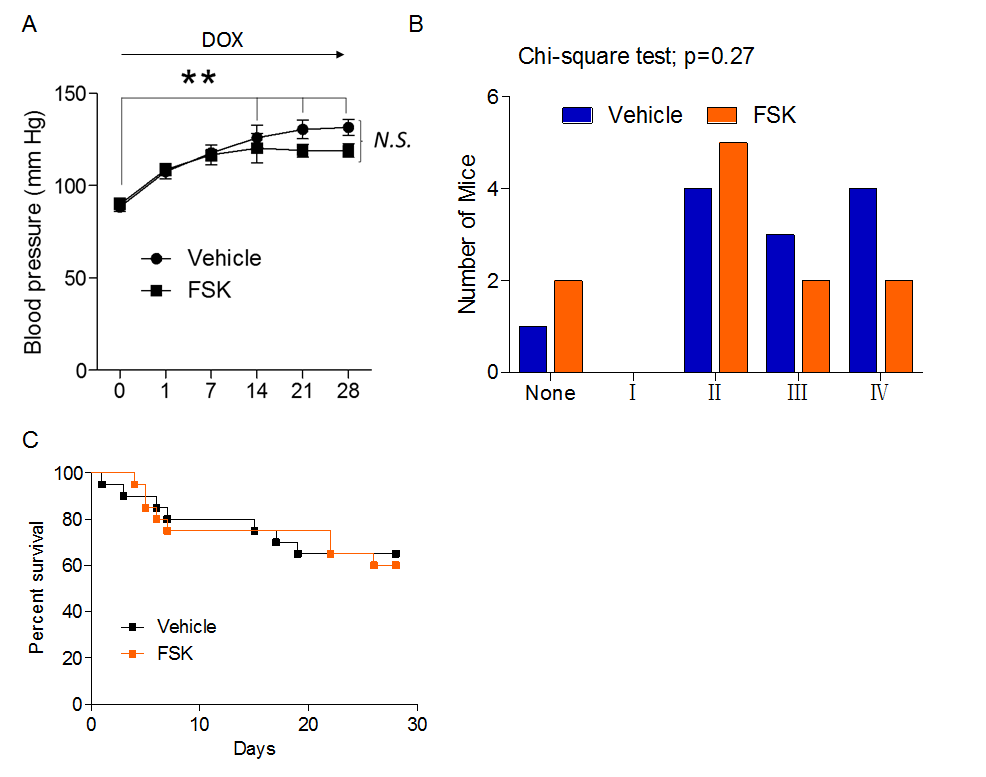


**Supplemental Figure 4. Null effect of FSK on blood pressure, abdominal aortic aneurysm (AAA) occurrence, and survival curve in mice with both hypertension and dyslipidemia. A**: DOX treatment increased blood pressure non-distinguishably between mice treated with FSK and vehilce. **B**: FSK did not affect the occurrence and classification of AAA. **C**: FSK treatment did not affect survival rates. n=8 (Vehicle), 10 (FSK) in A; n=12 (Vehicle), 11 (FSK) in B; n=20 in C. **p<0.01; ANOVA with repeated measured in A; Chi-square test in B and C.


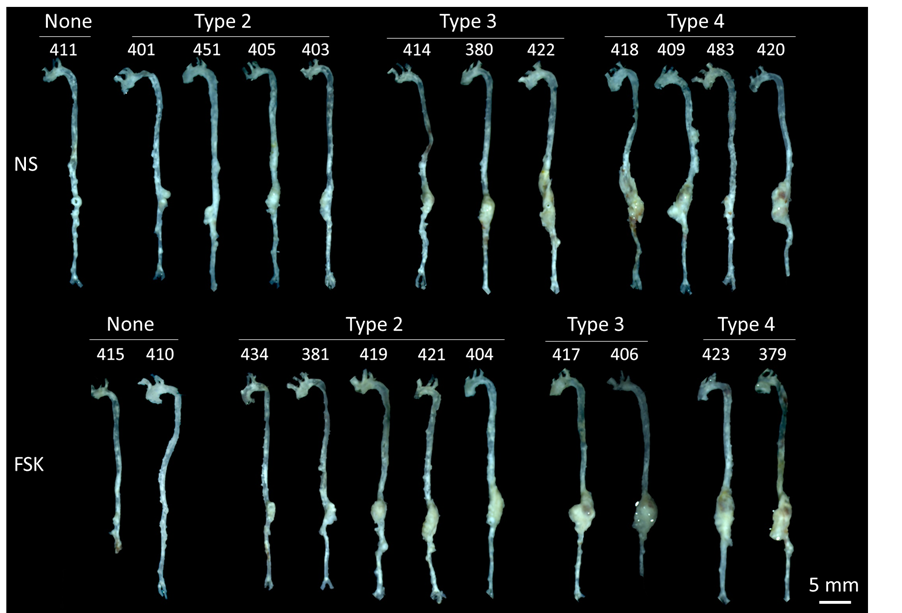


**Supplemental Figure 5. Images of the aortas showing the occurrence and classification of AAA.**


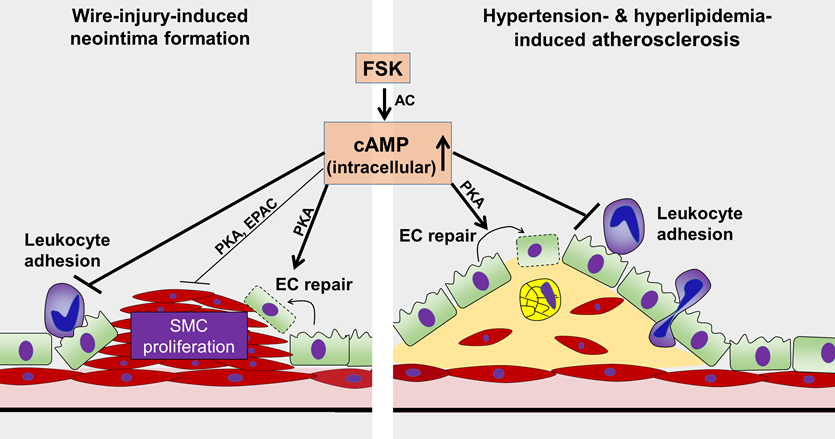
 **Supplemental Figure 6. Mechanistic illustration of the effect of FSK on wire-injury induced neointima formation (left panel) and atherosclerosis (right panel).** FSK elevates cAMP levels by activating AC (adenylate cyclase) and protects against neointima formation and atherosclerosis. Mechanistically, FSK differently regulates the proliferation of endothelial cells (ECs) and vascular smooth muscle cells (SMCs) and reduces leukocyte-endothelial interactions. FSK promotes endothelial repair by activating PKA, while it moderately suppresses SMC proliferation in both PKA- and EPAC- (exchange protein activated by cAMP) dependent manner.

**Supplemental Table：**

**Supplemental Table. Plasma lipid levels and body weight in ApoE^SA/SA^ mice treated with vehicle or forskolin (FSK) for 28 days. TG, Triglyceride；CHOL, Cholesterol；HDL-C，High density lipoprotein-cholesterol；LDL-C，Low density lipoprotein-cholesterol；BDW, Body weight. n=5-9.**

|  | **Vehicle** | **FSK** | **P Value** | **Significant?** |
| --- | --- | --- | --- | --- |
| **TG** | 2.14±0.21 | 1.58±0.07 | 0.06 | No |
| **CHOL** | 28.43±1.4 | 26.12±1.22 | 0.29 | No |
| **HDL-C** | 10.9±0.4 | 10.63±0.29 | 0.12 | No |
| **LDL-C** | 34.42±1.62 | 30.54±1.55 | 0.61 | No |
| **BDW** | 21.17±0.73 | 21.64±0.52 | 0.62 | No |

**Legends for Vedio files:**

**Effects of forskolin (FSK) administration on the rolling and adhesion of leukocytes in the femoral veins.** Vehicle (N) or FSK (F; 2mg/Kg) was intraperitoneally administrated 5 minutes before LPS infusion (5 mg/Kg/h). The Roman numerals denote the time point when the images were taken. The time point for starting LPS infusion was set to be 0 min.
